# Treatment of Methicillin-resistant *Staphylococcus aureus* experimental Osteomyelitis with bone-targeted Vancomycin

**DOI:** 10.1186/2193-1801-2-329

**Published:** 2013-07-18

**Authors:** Melissa J Karau, Suzannah M Schmidt-Malan, Kerryl E Greenwood-Quaintance, Jayawant Mandrekar, Jian Cai, William M Pierce, Kevyn Merten, Robin Patel

**Affiliations:** Division of Clinical Microbiology, Department of Laboratory Medicine and Pathology, Mayo Clinic College of Medicine, 200 First St. SW, Rochester, MN 5905 USA; Biomedical Statistics and Informatics, Department of Health Sciences Research, Mayo Clinic College of Medicine, Rochester, MN USA; Division of Infectious Diseases, Department of Medicine, Mayo Clinic College of Medicine, Rochester, MN USA; Department of Pharmacology and Toxicology, University of Louisville, Louisville, KY USA; Pradama Inc, Louisville, KY USA

**Keywords:** Vancomycin, Experimental osteomyelitis, MRSA, BT2-peg2-vancomycin

## Abstract

**Introduction:**

Methicillin-resistant *S. aureus* (MRSA) is a common cause of bone and joint infection. BT2-peg2-vancomycin is an investigational bone-targeted formulation of vancomycin which we hypothesized would have increased antimicrobial activity compared to conventional vancomycin in a chronic experimental MRSA osteomyelitis model.

**Methods:**

We tested bone affinity using an hydroxyapatite (HA) binding assay, assessed the *in vitro* antimicrobial susceptibility of 30 MRSA isolates, and compared vancomycin and BT2-peg2-vancomycin in a rat experimental osteomyelitis model.

**Results:**

Vancomycin did not bind to hydroxyapatite (HA binding index = 0), whereas BT2-peg2-vancomycin showed appreciable binding (HA binding index = 57). The MIC_50_ was 1 μg/ml and the MIC_90_ was 2 μg/ml for both vancomycin and BT2-peg2-vancomycin. The MBC_90_ was 16 and 4 μg/ml for vancomycin and BT2-peg2-vancomycin, respectively. Treatment with 50 mg/kg of vancomycin every 12 hours (median, 4.73 log_10_ cfu/g), 63.85 mg/kg (equivalent to 50 mg/kg vancomycin) of BT2-peg2-vancomycin every 12 hours (median, 3.93 log_10_ cfu/g) or 63.85 mg/kg of BT2-peg2-vancomycin once per week (median, 5.00 log_10_ cfu/g) was more active than no treatment (median, 5.22 log_10_ cfu/g) (P =0.0481). Treatment with 63.85 mg/kg of BT2-peg2-vancomycin every 12 hours was more active than all other treatment regimens evaluated (P≤0.0150), but was associated with high plasma BT2-peg2-vancomycin levels, decreased animal weight, increased kidney size, creatinine and BUN, and leukocytosis with tubulointerstitial nephritis.

**Conclusion:**

With optimization of pharmacokinetic parameters to prevent toxicity, BT2-peg2-vancomycin may be useful in the treatment of MRSA osteomyelitis.

## Introduction

Treatment of methicillin-resistant *Staphylococcus aureus* (MRSA) osteomyelitis usually requires surgical débridement and antimicrobial therapy. Vancomycin is often used for the latter; optimizing systemic vancomycin dosing may be challenging. Since osteomyelitis is a focal infection, local delivery of antimicrobial therapy may be useful. This has been done with local delivery of antimicrobial-impregnated polymethylmethacrylate. An alternative approach would be to formulate the antimicrobial agent such that, when delivered systemically, it targets bone.

Tetracycline binds hydroxyapatite and has been previously conjugated to other drugs, including estradiol; its complex structure and poor stability during chemical conjugation limit its use as a conjugated osteotropic agent. However, its hydroxyapatite binding domain could be specifically exploited to develop a bone-targeting agent. Using this strategy, bone-targeted estrogen has been developed (Neale et al. 2009).

**Figure 1:**
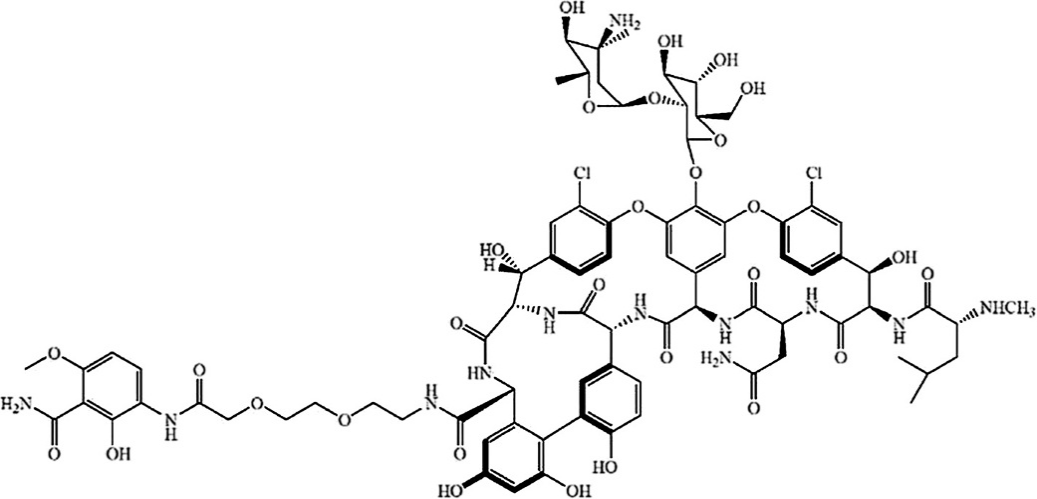
**Structure of BT2-peg2-vancomycin (C**_**80**_ **H**_**94**_ **Cl**_**2**_ **N**_**12**_ **O**_**29**_ **, molecular weight: 1758.57 g/mol) (Pierce et al.** 2011 **).**

Chemical conjugation of vancomycin to a bone-targeting moiety would theoretically yield a compound targeting delivery of vancomycin to bone, a strategy that could be useful in the treatment of bone infection. Pradama Inc. has developed a bone-targeted vancomycin, BT2-peg2-vancomycin (Figure 1) (Pierce et al. 2011). Previous studies show concentrations of BT2-peg2-vancomycin twelve hours after a single injection (63.85 mg/kg, equivalent to 50 mg/kg vancomycin) were 27.97 and 14.18 μg/ml for plasma and bone, respectively. Concentrations of BT2-peg2-vancomycin after seven doses given every 12 hours were 48.21 and 75.49 μg/ml for plasma and bone, respectively (Schmidt et al. 2011). The purpose of this study was to compare the *in vitro* and *in vivo* activity of BT2-peg2-vancomycin with that of conventional vancomycin.

## Materials and methods

### *In vitro*studies

#### Antimicrobial susceptibility

Thirty clinical isolates of MRSA were studied. The minimum inhibitory concentration (MIC) of oxacillin (Sigma-Aldrich, St. Louis, MO), vancomycin HCl (Sigma-Aldrich), and BT2-peg2-vancomycin diacetate containing 82.4% vancomycin (Pradama Inc., Louisville, KY) were determined using broth microdilution according to Clinical and Laboratory Standards Institute (CLSI) guidelines (Clinical and Laboratory Standards Institute 2012; Clinical and Laboratory Standards Institute 2013). The minimum bactericidal concentration (MBC) of vancomycin HCl and BT2-peg2-vancomycin diacetate were also determined according to CLSI guidelines (Clinical and Laboratory Standards Institute 1999).

#### Hydroxyapatite (HA) binding assay

10^-5^ M solutions of tetracycline, vancomycin HCl or BT2-peg2-vancomycin diacetate in 50 mM Tris–HCl buffer, pH 7.4, 1% DMSO were prepared. Tetracycline was used as a reference analyte; at 10^-5^ M, approximately 50% was bound to HA. An HA slurry of 0.5 g HA/100 ml 50 mM Tris–HCl buffer, 1% DMSO was prepared. For each analyte, two samples were tested in triplicate. For one sample, 1 ml of 10^-5^ M analyte and 100 μl 50 mM Tris–HCl buffer, 1% DMSO was pipetted into a microcentrifuge tube. For the second sample, 1 ml of 10^-5^ M analyte and 100 μl of the HA slurry was pipetted into a microcentrifuge tube. The samples were mixed gently by inversion for five minutes and then centrifuged at 12,000 g for three minutes to sediment the HA. The supernatant was transferred to another microcentrifuge tube. An electronic spectral scan (ultraviolet–visible) from 220–520 nm was obtained for each analyte using a Varian Cary 300 Bio Scan (Agilent Technologies, Santa Clara, CA). The blank was 50 mM Tris–HCl buffer, 1% DMSO. The wavelength of maximum absorbance (λ_max_) was determined, and the extinction coefficient (ϵ) calculated using the Beer-Lambert law. Absorbance of the samples incubated with HA was measured at λ_max_, and the molar concentration of the analyte determined using the Beer-Lambert law and the previously calculated extinction coefficient. The fraction adsorbed to HA was calculated for each sample. The HA binding capacity of each analyte was normalized to tetracycline by calculating the HA binding index, defined as percentage of analyte bound to HA/percentage of tetracycline bound to HA × 100 (Neale et al. 2009).

### *In vivo*studies

#### Experimental osteomyelitis

The experimental model described was performed in accordance with the guidelines of the Institutional Animal Care and Use Committee of the Mayo Clinic. Experimental osteomyelitis was established in 90 male Wistar rats using a modification of Zak’s model of experimental osteomyelitis (O'Reilly & Mader 1999). In earlier studies by our laboratory, we found that this model was highly reproducible with histological changes similar to those observed in humans with chronic osteomyelitis (Patel et al. 2000; Rouse et al. 2006). Animals were anesthetized with ketamine and xylazine and the proximal third of the medial left tibia surgically exposed. A 0.5 mm hole was drilled into the medullary cavity. Fifty microliters of morrhuate sodium (a sclerosing agent) was injected into the cavity, followed by 50 μl sterile saline containing 10^7^ colony forming units (cfu) of MRSA IDRL-4293. The muscle was reattached with coated vicryl sutures (Ethicon, Somerville, NJ). The skin was closed with tissue glue (Tissuemend, Butler Animal Health Supply, Dublin, OH) and wound clips (Harvard Apparatus, Holliston, MA). The wound was sprayed with a wound adhesive (Aluspray, Butler Animal Health Supply, Dublin, OH), along with a chewing repellent (Chewguard, Summit Hill Laboratories, Tinton Falls, NJ).

Four weeks after establishing infection, five rats were sacrificed, the left tibia aseptically removed and quantitative cultures performed. The remaining animals underwent surgical débridement of the infection site with sterile saline and were arbitrarily assigned to one of five study arms for a duration of 21 days: No treatment (n=17), vancomycin HCl every 12 hours (n=17, 42 doses), BT2-peg2-vancomycin diformate every 12 hours (n=17, 42 doses), BT2-peg2-vancomycin diformate every 12 hours for 3.5 days and then every fourth day thereafter (n=16, 11 doses), and BT2-peg2-vancomycin diformate once per week (n=15, 3 doses). Vancomycin HCl was administered at 50 mg/kg and BT2-peg2-vancomycin diformate at 63.85 mg/kg (equivalent to 50 mg/kg active vancomycin); both were administered intraperitoneally. Twelve hours after completion of therapy (day 50), blood was collected via cardiac puncture for hematology and chemistry studies [performed by Charles River (Wilmington, MA)], and drug concentration studies. Rats were sacrificed with CO_2_. The left (infected) tibia from one animal in each group was collected for histopathology with bones placed in 10% neutralized buffered formalin (NBF), decalcified, embedded in parafilm, prepared as longitudinal cuts and stained with hematoxylin and eosin. The left (infected) tibias from the remaining animals were weighed, cryopulverized and processed for quantitative and qualitative bacterial culture. Culture results were expressed as log_10_ cfu/g of bone tissue.

Ten MRSA colonies per animal recovered from quantitative bacterial cultures were tested for susceptibility to BT2-peg2-vancomycin diformate and/or vancomycin HCl.

Kidneys from all animals were weighed, observed for abnormalities in size or color, and placed in NBF. Histological analysis was performed by Seventh Wave (Chesterfield, MO). Microscopic lesions were graded on a scale of one to four, with four being the most severe. Tubulointerstitial nephritis was characterized by multifocal non-suppurative interstitial inflammation, interstitial fibrosis, tubular epithelial degeneration, necrosis, and regeneration with frequent intra-tubular neutrophilic inflammation and cellular debris.

Three right tibias from each group were collected, weighed, placed into NBF, and sent to Histion LLC (Everett, WA). They underwent decalcification and were embedded in paraffin, cut into three frontal sections, and stained with either Masson’s trichrome, hematoxylin and eosin, or toluidine blue. Sections were sent to Think Bone Consulting (Seattle, WA) for histomorphometry performed using an OsteoMeasureXP system (Osteometrics, Inc., Atlanta, GA). Bone mass and possible toxic effects were assessed by measuring tissue area (bone/marrow), bone area and perimeter, and perimeter of bone lined by osteoblasts.

Plasma concentrations of BT2-peg2-vancomycin in infected animals were measured by liquid chromatography/mass spectrometry (LC/MS). Plasma samples (50 μl) were mixed with 200 μl of 25 μg/ml teicoplanin (Sigma, St. Louis, MO) in 1% formic acid. Proteins were precipitated with 750 μl of acetonitrile (ACN) and centrifuged at 13,000 RPM for two minutes. The supernatant was collected and concentrated to 50 μl by speedvac, diluted with 100 μl 5% ACN in 0.1% formic acid, loaded onto a prewashed C_18_ spin column (Nest Group, Inc, Southborough, MA), washed three times with 100 μl 5% ACN in 0.1% formic acid, and eluted twice with 100 μl 5% ACN in 0.1% formic acid. The elutes (50 μl) were diluted with 450 μl 5% ACN in 0.1% formic acid and analyzed by Accela LC System (Thermo Scientific, San Jose, CA) coupled with a LTQ-Orbitrap XL mass spectrometer (Thermo Scientific, San Jose, CA). Samples were loaded onto a 50 × 2.1 mm × 1.9 μm Hypersil GOLD column (Thermo Scientific, San Jose, CA) and eluted with a 17 minute binary solvent gradient (solvent A: 5% ACN/0.1% formic acid and solvent B: 95% ACN/0.1% formic acid) at 100 μl/minute. The gradient started from 5% solvent B, increased linearly to 80% solvent B in 12 minutes, and then remained at 80% B for 5 minutes. Elutes from the LC column were ionized by electrospray ionization and BT2-peg2-vancomycin and teicoplanin were detected by multiple reaction monitoring. Full scan fragment spectra from precursor ions (double charged m/z 880.3 from 5.5 to 7 minutes for BT2-peg2-vancomycin and double charged m/z 940.8 from 7 to 10 minutes for teicoplanin) were acquired by orbitrap. Compound confirmation was obtained from fragment spectra and concentration was calculated from peak areas of fragment ion chromatograms (m/z 1614.47 at 6.25 minutes for BT2-peg2-vancomycin diformate and m/z 1563.36 at 7.95 minutes for teicoplanin) and a calibration curve from control rat plasma (Biochemed Services Winchester, VA) spiked with authentic BT2-peg2-vancomycin diformate (0 to 200 μg/ml).

Plasma concentrations of vancomycin were measured by LC/MS. Protein precipitation and C_18_ spin column cleanup were performed as described for BT2-peg2-vancomycin diformate except that the teicoplanin concentration was 2.5 μg/ml and 0.1% formic acid was used as washing solvent. Elutes from the C_18_ column were concentrated to approximately 10 μl by speedvac, diluted to 100 μl with 0.1% formic acid, and analyzed by LC/MS with same column, gradient and instruments. Full scan fragment spectra from precursor ions (double charged m/z 725.7 from 0 to 5.5 minutes for vancomycin and double charged m/z 940.8 from 7 to 10 minutes for teicoplanin) were acquired by orbitrap. Compound confirmation was obtained from fragment spectra and concentration was calculated from peak areas of fragment ion chromatograms (m/z 1305.34 at 5.0 minutes for vancomycin and m/z 1563.36 at 7.95 minutes for teicoplanin) and a calibration curve from control rat plasma spiked with vancomycin standard (0 to 5 μg/ml).

### Statistical methods

Descriptive summaries for bacterial culture, hematology and chemistry studies are reported as medians and ranges. Comparisons between groups were performed using the Kruskall Wallis test. If the overall test among the groups was significant, further pairwise comparisons were made using the Wilcoxon rank-sum test. Given the small sample sizes, no adjustments for multiple comparisons were made. All tests were two sided and p-values less than 0.05 were considered statistically significant. Statistical analysis was performed using SAS version 9.2 (SAS Inc., Cary, NC).

## Results

### *In vitro*studies

#### Antimicrobial susceptibility

All isolates were resistant to oxacillin, with a MIC range of 4 to >128 μg/ml. The oxacillin MIC for 50% of the isolates was >128 μg/ml. The MIC at which 50% of the isolates were inhibited (range) was 1 μg/ml (0.5 to 2 μg/ml) for both vancomycin HCl and BT2-peg2-vancomycin diacetate. The MBC at which 50% of isolates were killed (range) was 2 μg/ml (0.5 to 64 μg/ml) for vancomycin HCl and 2 μg/ml (0. 5 to >128 μg/ml) for BT2-peg2-vancomycin diacetate. The MIC at which 90% of the isolates were inhibited was 2 μg/ml for both vancomycin HCl and BT2-peg2-vancomycin diacetate. The MBC at which 90% of isolates were killed was 16 and 4 μg/ml for vancomycin HCl and BT2-peg2-vancomycin diacetate, respectively.

#### HA binding assay

The bone-targeting capacity of vancomycin HCl and BT2-peg2-vancomycin diacetate was evaluated *in vitro* using an HA binding assay. For comparative purposes tetracycline was included as a control and was defined as having an HA binding index of 100. Vancomycin HCl did not bind to HA (HA binding index = 0), whereas BT2-peg2-vancomycin diacetate showed appreciable binding to hydroxyapatite (HA binding index = 57). This suggests that vancomycin does not attach to HA but that it is the bone-targeting moiety which is attached to the parent vancomycin molecule by polyethelene glycol linkage (Figure 1) which is responsible for BT2-peg2-vancomycin’s ability to bind to the mineral constituent of bone.

### *In vivo*studies

#### Experimental osteomyelitis

The MIC/MBC of the MRSA isolate used in the experimental osteomyelitis model was 2/2 μg/ml for both vancomycin HCl and BT2-peg2-vancomycin diformate. Three of the 85 rats did not survive the débridement process. Five were excluded due to *Proteus* sp. culture contamination and one due to procedure error. The five control rats showed a median of 5.56 (range, 5.25 to 5.93) log_10_ cfu/g of bone after the 28 day infection period.

**Figure 2:**
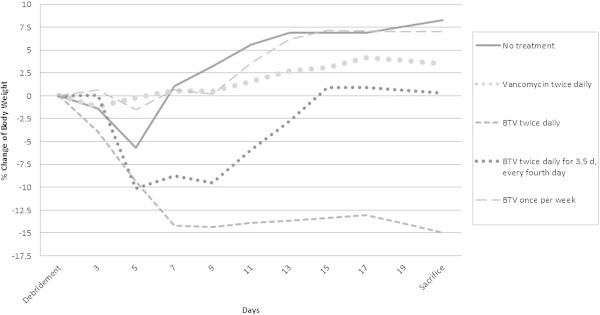
**Percent body weight change over the 21 day course of treatment.** The difference between BT2-peg2-vancomycin diformate (BTV) administered every 12 hours and each of the other treatment groups was statistically significant (P<0.0001) at time of sacrifice.

The percent change in body weight over the course of 21 days of treatment for each treatment group is shown in Figure 2. The terminal body weights of the animals treated with BT2-peg2-vancomycin diformate every 12 hours were statistically lower than those of each of the other treatment groups (P<0.0001). The bone specimens reviewed from one animal in each group were confirmed to have histological features consistent with osteomyelitis (Vigorita 2008). An acute inflammatory response along with microabscess formation was observed in all specimens.

**Figure 3:**
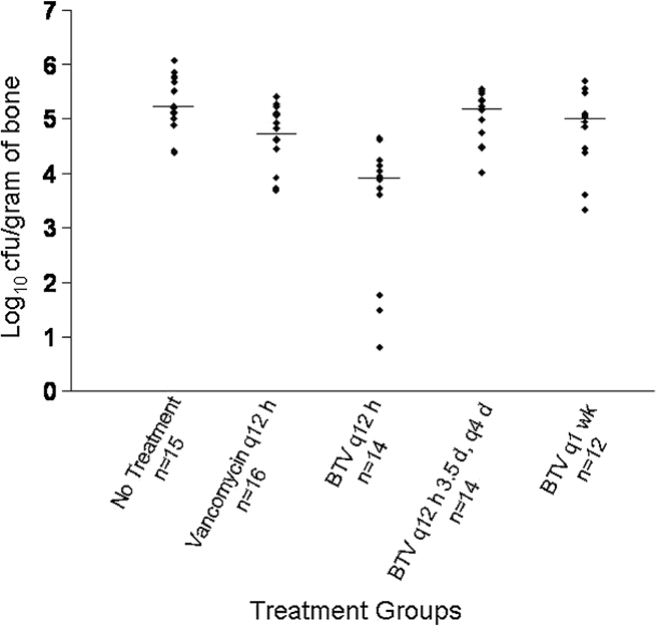
**Results of the quantitative bone cultures showing median values for each treatment group.** The difference between BT2-peg2-vancomycin diformate (BTV) every 12 hours (q12 h) and each of the other groups was statistically significant (P ≤ 0.0150). (BTV q12 h 3.5 d, q4 d, treatment twice daily for 3.5 days and then once every 4 days; BTV q1 wk, treatment once per week).

Results of quantitative cultures are shown in Figure 3. The untreated group had a median of 5.22 (range, 4.37 to 6.08) log_10_ cfu/g of bone. The vancomycin HCl group had a median of 4.73 (range 3.69 to 5.41) log_10_ cfu/g of bone. The median was 3.93 (range, 0.80 to 4.66) log_10_ cfu/g of bone in the BT2-peg2-vancomycin diformate every 12 hours group, 5.19 (range, 4.02 to 5.54) log_10_ cfu/g of bone in the BT2-peg2-vancomycin diformate every 12 hours for 3.5 days then every fourth day group, and 5.00 (range, 3.33 to 5.70) log_10_ cfu/g of bone in the BT2-peg2-vancomycin diformate once per week group. The bacterial loads in the animals treated with vancomycin HCl twice daily, BT2-peg2-vancomycin diformate twice daily and once weekly were significantly lower than those in the untreated animals (P=0.0037, P<0.0001, P=0.0481 , respectively). The bacterial load in the animals treated with BT2-peg2-vancomycin diformate twice daily was lower than in the no treatment, twice daily vancomycin, BT2-peg2-vancomycin diformate every 12 hours for 3.5 days then every fourth day and BT2-peg2-vancomycin once weekly groups (P<0.0001, P=0.0150, P<0.0001 and P=0.0034, respectively). Vancomycin MIC values of the MRSA recovered from the untreated group were 1 to 2 μg/ml and from the vancomycin HCl-treated animals were 2 to 4 μg/ml. Isolates recovered from BT2-peg2-vancomycin diformate-treated animals were tested for susceptibility to both vancomycin and BT2-peg2-vancomycin. Isolates from those treated with BT2-peg2-vancomycin diformate twice daily had vancomycin and BT2-peg2-vancomycin MICs of 1 to 4 μg/ml. Isolates from those treated with BT2-peg2-vancomycin diformate twice daily for 3.5 days and then once every fourth day had vancomycin MICs of 1 to 2 μg/ml and BT2-peg2-vancomycin MICs of 1 to 4 μg/ml. Isolates from those treated with BT2-peg2-vancomycin diformate once weekly had vancomycin and BT2-peg2-vancomycin MICs of 1 to 2 μg/ml. No increase in vancomycin or BT2-peg2-vancomycin MIC of more than one doubling dilution was observed.

Bone histology/histomorphometry of the three right tibias from each group showed no changes in bone mass of the treated *versus* the untreated animals (P<0.05). Myelosuppression was not observed in any treatment group.

**Figure 4:**
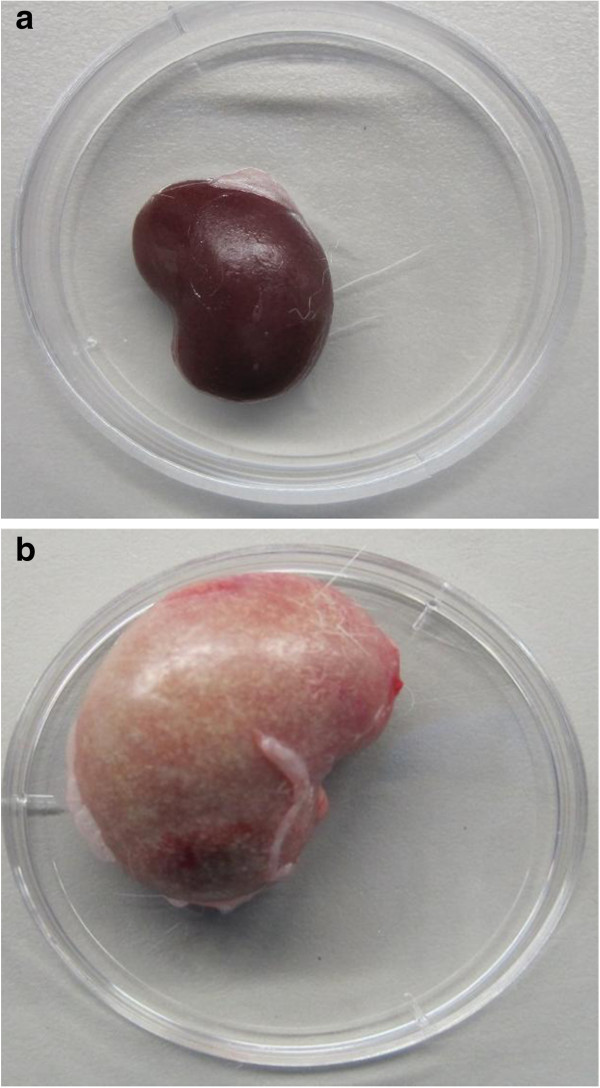
**a. Kidney following no treatment (~19.0 × 12.5 mm); b. kidney following treatment with BT2-peg2-vancomycin diformate twice daily for 21 days (~25.5 × 17.5 mm).**

The kidneys of the untreated and the vancomycin HCl-treated animals had a deep maroon color and size consistent with body weight. The kidneys from the group given BT2-peg2-vancomycin diformate every 12 hours for 21 days were lighter in color and larger than those of the animals in the other treatment groups (Figure 4). Kidney weights are shown in Table 1. Tubulointerstitial nephritis was observed in animals treated with BT2-peg2-vancomycin diformate and increased in incidence and severity with increasing frequency of administration of BT2-peg2-vancomycin diformate. In the group given BT2-peg2-vancomycin diformate every 12 hours, the findings were histologically severe (grade 4) and were associated with azotemia [increased creatinine and blood urea nitrogen (BUN)] and leukocytosis. In the group given BT2-peg2-vancomycin diformate twice daily for 3.5 days and every fourth day thereafter, the histology showed moderate tubulointerstitial nephritis (grades 2–4) and the once weekly-treated group had mild tubulointerstitial nephritis (grades 1–2). The kidneys from the untreated and vancomycin HCl-treated groups did not exhibit microscopic abnormalities.

**Table 1:** **Kidney weights, creatinine, BUN and albumin (range) values after treatment with vancomycin HCl or BT2-peg2-vancomycin diformate (BTV)**

Group	Kidney weight (grams)	Creatinine (mg/dl)	BUN (mg/dl)	Albumin (g/dl)
No treatment	1.4 (1.2-1.9)	0.5 (0.5-0.6)	20 (17–25)	3.6 (3.3-3.9)
Vancomycin HCl twice daily	1.4 (1.1-1.7)	0.5 (0.4-0.6)	18.5 (15–21)	3.4 (3.1-3.6)
BTV twice daily	4.1 (2.4-6.0)	1.5 (1.2-2.2)	76.5 (56–114)	2.8 (2.6-3.0)
BTV twice daily for 3.5 days and then every four days	1.7 (1.3-2.2)	0.6 (0.5-1.2)	31 (22–47)	3.6 (3.1-4)
BTV once per week	1.5 (1.4-1.8)	0.5 (0.4-0.6)	19 (17–21)	3.6 (3.3-3.8)

In the group given BT2-peg2-vancomycin diformate every 12 hours, the median creatinine (range) was elevated at 1.5 (1.2 to 2.2) mg/dl (normal creatinine range 0.4 to 0.7 mg/dl), the median (range) BUN was elevated at 77 (56 to 114) mg/dl (normal BUN 9–30 mg/dl) and the median (range) serum albumin was decreased at 2.8 (2.6 to 3.0) g/dl (normal range 3.2 to 4.5 g/dl) (Table 1). Creatinine, BUN, and albumin were statistically significantly different in this group compared to all the other groups (P<0.0001). In the group given BT2-peg2-vancomycin diformate twice daily for 3.5 days and every fourth day thereafter, the median (range) creatinine and BUN were 0.6 (0.5 to 1.2) and 31 (22 to 47) mg/dl which was higher than that in the no treatment, vancomycin HCl-treated or BT2-peg2-vancomycin diformate once weekly-treated animals (P<0.0001).

In the group given BT2-peg2-vancomycin diformate every 12 hours, the median (range) total white cell count was 34.75 (25.04 to 49.82, normal range 3.66 to 16.05) × 10^3^/μl, neutrophil count was 20.89 (16.92 to 30.05, normal range 1.02 to 6.63) × 10^3^/μl, eosinophil count was 1.13 (0.26 to 10.92, normal range 0.01 to 0.51) × 10^3^/μl, and basophil count was 0.21 (0.06 to 0.41, normal range 0.00 to 0.15) × 10^3^/μl, all higher than in the other groups (P<0.0001).

The median (range) plasma concentration of vancomycin 12 hours after the last injection in the twice daily vancomycin HCl treated animals was 0.71 (0.24 to 2.00) μg/ml (Table 2). The median (range) plasma concentration of BT2-peg2-vancomycin 12 hours after the last injection in the twice daily BT2-peg2-vancomycin diformate treated animals was 129.98 (74.56 to 207.90) μg/ml. The median (range) plasma concentration of BT2-peg2-vancomycin 48 hours after the last injection in the animals treated with BT2-peg2-vancomycin diformate every 12 hours for 3.5 days and every fourth day thereafter was 0.88 (0.00 to 4.14) μg/ml. The median (range) plasma concentration of BT2-peg2-vancomycin seven days after the last injection in the animals treated once per week with BT2-peg2-vancomycin diformate was 0.00 (0.00 to 0.34) μg/ml.

**Table 2:** **Plasma concentrations (μg/ml) of vancomycin or BT2-peg2-vancomycin (BTV) in the osteomyelitis animals following 21 days of treatment**

	n	Plasma concentration (μg/ml)		Time (h) after last dose
		Median	Range	
Vancomycin HCl twice daily	16	0.71	0.24-2.00	12
BTV twice daily	13	129.98	74.56-207.90	12
BTV twice daily for 3.5 days and then every four days	14	0.88	0.00-4.14	48
BTV once per week	15	0.00	0.00-0.34	168

## Discussion

BT2-peg2-vancomycin is a novel formulation of vancomycin designed to target bone. We have demonstrated that it has similar *in vitro* activity to vancomycin against MRSA, indicating that BT2-peg2 formulation does not affect the antimicrobial activity of vancomycin. We have additionally shown that the addition of the bone-targeting moiety to vancomycin enhances its *in vitro* ability to bind to the mineral constituent of bone. Results of pharmacokinetic studies also support targeting of bone; however, this is not without presence of BT2-peg2-vancomycin in plasma.

Results of treatment of experimental osteomyelitis with equivalent amounts of vancomycin at the same dosing interval show that BT2-peg2-vancomycin diformate is more active than vancomycin. However, this dosing schedule of BT2-peg2-vancomycin diformate is associated with high plasma BT2-peg2-vancomycin levels and toxicity, manifest as decreased animal weight, and increased kidney size with increased creatinine and BUN, and leukocytosis in the context of histopathologic severe tubulointerstitial nephritis. The high serum concentrations of BT2-peg2-vancomycin likely relates to pegylation of the parent drug which is known to increase drug half-life (Hamidi et al. 2006), and may alter elimination of drug from the body and biodistribution. Alternatively, the high serum BT2-peg2-vancomycin levels may be caused by binding, retention and/or subsequent release of BT2-peg2-vancomycin from the bone over time. In experimental osteomyelitis, BT2-peg2-vancomycin diformate administered weekly yielded similar results to twice daily doses of vancomycin, without the toxicity observed with twice daily dosing.

A limitation of this study is the dosing interval of vancomycin HCl was selected to match that of BT2-peg2-vancomycin, resulting in low vancomycin trough concentrations in the vancomycin HCl treated animals. Ideally, vancomycin HCl should have been dosed more frequently to mimic in human levels.

Overall, results of this study indicate that BT2-peg2-vancomycin deserves further evaluation as a bone-targeted formulation of vancomycin with less frequent dosing required than conventional vancomycin, and careful assessment of plasma vancomycin levels and associated toxicity required.

## References

[CR1] Clinical and Laboratory Standards Institute (1999). Methods for determining bactericidal activity of antimicrobial agents; approved guideline. CLSI document M26-A vol 19.

[CR2] Clinical and Laboratory Standards Institute (2012). Methods for dilution antimicrobial susceptibility tests for bacteria that grow aerobically; Approved Standard-Ninth Edition CLSI document M07-A9, vol 32.

[CR3] Clinical and Laboratory Standards Institute (2013). Performance standards for antimicrobial susceptibility testing; Twenty-ThirdInformational Supplement M100-S23.

[CR4] Hamidi M, Azadi A, Rafiei P (2006). Pharmacokinetic consequences of pegylation. Drug Deliv.

[CR5] Neale JR, Richter NB, Merten KE (2009). Bone selective effect of an estradiol conjugate with a novel tetracycline-derived bone-targeting agent. Bioorg Med Chem Lett.

[CR6] O'Reilly T, Mader J, Zak O, Sande MA (1999). Rat model of bacterial osteomyelitis of the tibia. Handbook of animal models of infection.

[CR7] Patel R, Piper KE, Rouse MS, Steckelberg JM (2000). Linezolid therapy of *Staphylococcus aureus* experimental osteomyelitis. Antimicrob Agents Chemother.

[CR8] Pierce W, Taylor K, Waite L (2011). Methods and compounds for the targeted delivery of agents to bone for interaction therewith.

[CR9] Rouse MS, Piper KE, Jacobson M, Jacofsky DJ, Steckelberg JM, Patel R (2006). Daptomycin treatment of *Staphylococcus aureus* experimental chronic osteomyelitis. J Antimicrob Chemother.

[CR10] Schmidt SM, Albayati ZF, Karau MJ (2011). Rat plasma and bone concentrations of BT2-peg2-vancomycin and vancomycin via intraperitoneal administration.

[CR11] Vigorita VJ (2008). Orthopaedic pathology.

